# Associations between Dietary Antioxidant Vitamin Intake and the Changes in Bone Mass in Chinese Adolescents: A 2.5-Year Longitudinal Study

**DOI:** 10.3390/nu14194187

**Published:** 2022-10-08

**Authors:** Hui Li, Jin-Li Hou, Wan-Yu Yang, Qiong Zhang, Hao Feng, Xiao-Bao Wang, Kai-Li Deng, Xin-Liang Mao, Su-Mei Xiao

**Affiliations:** 1 Department of Epidemiology, School of Public Health, Sun Yat-sen University, Guangzhou 510080, China; 2 College of Food Science and Engineering, South China University of Technology, Guangzhou 510640, China; 3 Guangdong Provincial Key Laboratory of Food, Nutrition and Health, School of Public Health, Sun Yat-sen University, Guangzhou 510080, China

**Keywords:** bone mass, antioxidant vitamins, dietary, adolescents

## Abstract

(1) Background: Optimal bone mass accumulation during adolescence is crucial for maximising peak bone mass during adulthood. Dietary antioxidant vitamins may contribute to bone mass accumulation. This 2.5-year-long longitudinal study aimed to evaluate the relationships between dietary vitamin A, C, and E intakes and the annual changes in bone parameters among Chinese adolescents. (2) Method: Subjects aged 10–18 years (*n* = 1418) were recruited from a secondary school in Jiangmen, China. Dietary vitamin A, C, and E intakes were assessed using 24 h dietary records over 3 consecutive days. The Sahara Clinical Bone Sonometer was used to measure the broadband ultrasound attenuation (BUA) and the speed of sound (SOS). Their annual changes were then calculated (i.e., BUA%/year, SOS%/year). The associations were detected after adjusting for the baseline bone phenotype; age; sex; weight; height; pubertal stage; physical activity; and dietary intakes of vitamin D, calcium and energy. (3) Results: A curvilinear relationship was found between the dietary intake of vitamin C and BUA%/year (*p* = 0.026); further analyses in the subgroups revealed that this relationship was observed in male adolescents (*p* = 0.012). A positive association was observed only in boys with a dietary vitamin C intake of ≥159.01 mg/day (β = 0.395, *p* = 0.036). Moreover, a linear positive association was shown between the dietary intake of vitamin E and BUA%/year in female adolescents (β = 0.082, *p* = 0.033). (4) Conclusion: Our findings indicated that dietary vitamin C intake has a threshold effect on bone mass gain in male adolescents and that dietary vitamin E intake could be a positive predictor of bone mass gain in female adolescents.

## 1. Introduction

Peak bone mass (PBM) is a critical predictor of the lifetime risk of osteoporosis in middle-aged and old individuals [1,2]. The earliest age to achieve PBM is usually 17–18 years, and the latest is usually 35 years [1]. Every 10% increase in PBM reportedly delays the onset of osteoporosis by 13 years [2]. Studies have reported that approximately 40% of PBM is achieved during puberty and that by the end of puberty, PBM could reach >95% of its maximum value [3]. Thus, it is important to study the factors affecting bone mass accumulation during adolescence to maximise PBM and prevent osteoporosis during adulthood.

Antioxidant vitamins, namely, vitamin A, C, and E, can alleviate the harmful effects of oxidative stress (OS) on the bone. OS is a state of imbalance between oxidation and antioxidation and is caused by the excessive production of free radicals and reactive oxygen species (ROS) [4]. This is linked with the pathogenesis of various human diseases, including osteoporosis [4]. In vitro studies have reported that the over-generation of ROS induces osteoclast formation and bone resorption by stimulating the expression of receptor activator of nuclear factor-kappa B ligand and macrophage-stimulating factor [5,6]. Epidemiological data have also suggested that OS is an independent risk factor for bone loss and osteoporosis [6,7]. Individuals with osteoporosis or lower bone mass express significantly higher levels of biochemical markers for OS [6,7]; such individuals also exhibit a decline in their antioxidant capacity, as reflected in a drop in plasma vitamin A, C, and E levels and the activity of glutathione peroxidase and superoxide dismutase [4,8]. Studies have shown that antioxidant vitamin supplements can reduce the levels of OS and improve bone health [9,10,11]. Chavan et al. reported that osteoclast activity is inhibited and the body’s antioxidant status is improved among osteoporotic patients consuming vitamin C and/or E supplements [10]. Pasco et al. reported that the duration of vitamin C and/or E supplementation is inversely associated with the serum concentrations of bone turnover markers in postmenopausal women [11]. 

Studies on the effects of dietary antioxidant vitamins, namely vitamins A, C and E, on bone mass accumulation in adolescents are limited. Several cross-sectional studies conducted in adolescents have found that dietary intake of vitamin C is positively related to bone mass [12,13,14]. By contrast, a 10-year-long prospective cohort study involving 61 girls aged 9–11 years reported that dietary intake of vitamin C was not correlated with calcaneal bone ultrasound parameters [15]. Moreover, several studies have reported varied results regarding the relationship between dietary vitamin A intake and bone parameters in adolescents, with either positive [16] or insignificant [12] associations. Only one study has reported an association between dietary intake of vitamin E and bone parameters in adolescents, and the association was not significant [16]. 

Therefore, it is important to understand how vitamins A, C, and E affect bone mass accumulation during adolescence. Accordingly, we investigated the relationships of dietary vitamin A, C, and E intakes with the longitudinal rates of changes in bone parameters among Chinese adolescents followed up for 2.5 years; we also explored the sex-based differences.

## 2. Materials and Methods

### 2.1. Subjects

The study recruited students (aged 10–18 years) from a secondary school in Jiangmen, China. Recruitment proceeded through advertisements and invitation to attend the health talks held for each class at the school. Participation was voluntary. Students with past or current conditions affecting bone or mineral metabolism, such as cancer, thyroid disease, parathyroid disease, stomach ulcers, chronic kidney disorder, and glucocorticoid use, were to be excluded. However, no student was excluded because none of the students had any of these conditions. The baseline surveys were carried out between October and November 2015, and a total of 1472 students (774 female and 698 male students) were recruited. Finally, 1418 students (721 female and 697 male students) who completed the follow-up surveys between March and April 2018 were included as the study subjects. All subjects were followed for approximately 2.5 years. Prior to the study, written informed consent from all the subjects and their parents or legal guardians was collected. This study was approved by the Ethics Committee of Jiangmen Central Hospital and Affiliated Jiangmen Hospital of Sun Yat-sen University (protocol code (2015) 7). 

### 2.2. Calcaneal Quantitative Ultrasound Measurements

Calcaneal quantitative ultrasound (QUS) was measured with the Sahara Clinical Bone Sonometer (Hologic, Bedford, MA, USA) at the right heel. QUS estimates the bone status by evaluating ultrasound velocity and attenuation. Compared with the gold standard method, namely, dual-energy X-ray absorptiometry (DXA), QUS is portable, cost-effective, and radiation-free, making it particularly suitable for bone mass assessment among healthy children and adolescents in epidemiological studies [17]. Broadband ultrasonic attenuation (BUA; dB/MHz) and the speed of sound (SOS; m/s) were determined from the QUS measurements. BUA is an indicator of bone mineral density (BMD) and is closely related to BMD measured using DXA (r = 0.83) [18]. SOS reflects the bone microstructure and elasticity and is also associated with DXA-measured BMD (r = 0.66) [19]. In this study, two measurements were conducted per student to obtain the baseline and follow-up bone parameter values. The ultrasonic meter was subjected to quality control by placing the phantom directly into its sensor prior to measurement. The in vivo precision of BUA and SOS were 2.30% and 0.20%, respectively. 

### 2.3. Assessment of Dietary Vitamin A, C, and E Intakes

All the subjects were interviewed at baseline for dietary intake estimates using the 24 h dietary recall records over three consecutive days (including 2 weekdays and 1 weekend day). Pictures of the consumed food were used to document the type and quantity of food eaten. Dietary vitamin A, C, and E intakes were assessed using the Chinese Food Composition Table [20,21]. Dietary vitamin A intake was calculated in retinol activity equivalents (RAE) as follows: RAE = (intake of retinol (μg)) + {1/12 (intake of carotene (μg))}. Dietary intake of vitamin E was calculated in α-tocopherol equivalents (α-TE) as follows: α-TE = [intake of α-tocopherol (mg)) + {0.5 (intake of β-tocopherol (mg))} + {0.1 (γ-tocopherol (mg))} + {0.3 (trientocopherol (mg))} + {0.01 (δ-tocopherol (mg))}. The average daily dietary intakes of vitamin A, C and E were calculated and used in the analysis. Information about whether the subjects had taken vitamin and calcium supplements over the past year was also collected. Approximately 3.95% of the subjects (29 female and 27 male) took supplements, particularly multivitamins. Quantitative data about the supplement use were not available; thus, the assessment of the dietary intakes of the three studied vitamins were based on the subjects’ 24 h dietary reviews at baseline. 

### 2.4. Assessment of Covariates

Information regarding the subjects’ demographic characteristics, disease history, medications, and lifestyle habits was collected at baseline with a structured questionnaire administered during a face-to-face interview carried out by the trained staff. Weight and height were estimated by standardised equipment. Weight was evaluated with an accuracy of 0.1 kg without shoes or heavy clothing. Height was assessed to the nearest 0.1 cm without shoes. Physical activity was estimated by the modified Children’s Leisure Activities Study Survey Chinese version questionnaire [22] and expressed in metabolic equivalents (h/day). Dietary energy, vitamin D, and calcium intakes were also assessed by the 24-h dietary recall records over three consecutive days at baseline. Dietary energy and calcium intakes were calculated using the Chinese Food Composition Table [21]. Dietary vitamin D intake was evaluated using the Standard Tables of Food Composition in Japan [23]. The average daily dietary intakes of energy, vitamin D, and calcium were calculated and used in the analysis. The Chinese version of the self-reported Pubertal Development Scale, which assesses five items, including growth spurt, deepening voice, body hair growth, facial hair growth, and skin changes, was used to estimate the pubertal development stage [24]. The subjects were classified as early-mid pubertal (79 female and 244 male subjects) or late-post pubertal (642 female and 453 male subjects) using these data. 

### 2.5. Statistical Analysis

Continuous variables are presented as means and standard deviations. The sex-based differences in the parameters were examined using the independent-sample *t*-test. A generalised additive model (GAM) was first used to explore the functional form of the relationships of the dietary intakes of vitamin A, C, and E with the annual changes in bone parameters. The rates of the changes in bone parameters per year as the dependent variables were calculated as the changes in each bone parameter divided by the baseline value and the follow-up duration (i.e., BUA%/year and SOS%/year). GAM analysis was also performed in the sex-based subgroups. If linearity was observed in the GAM analysis, multiple linear regression analyses were used to further examine the association. If nonlinearity was observed in the GAM analysis, a two-piecewise multiple linear regression analysis [25] was conducted to determine the break-point of the associations between dietary vitamin A, C, and E intakes and the annual changes in bone parameters. The break-point was estimated as part of the model-fitting process by likelihood maximization. Confounding factors such as the baseline bone phenotype, age, weight, height, physical activity, dietary calcium intake, dietary vitamin D intake, dietary energy intake, pubertal stage, and/or sex were adjusted in the models. All data were analysed using R software (version 3.5.1; R Foundation for Statistical Computing, Vienna, Austria) and SPSS software (version 20.0; SPSS Inc., Chicago, IL, USA). A two-sided *p* value of <0.05 was considered statistically significant. 

## 3. Results

### 3.1. Descriptive Characteristics

The characteristics of the 1418 subjects (721 female and 697 male subjects) are shown in Table 1. The average age of the subjects was 14.97 ± 1.46 years. The height, weight, total physical activity, and dietary energy and calcium intakes were significantly higher among the male subjects than the female subjects (all *p* < 0.010). The mean dietary vitamin E intake was significantly higher *(p* < 0.001) in the female subjects (15.33 ± 8.48 mg/day) than in the male subjects (13.82 ± 8.74 mg/day). The overall mean dietary vitamin D, A, and C intakes were 1.85 ± 1.29 µg/day, 994.10 ± 528.45 µg RAE/day, and 82.57 ± 47.37 mg/day, respectively. No significant between-sex differences were noted (all *p* > 0.050) for them. The relative rate of changes in BUA%/year was significantly greater (*p* = 0.002) in the male subjects (22.13%/year) than in the female subjects (18.92%/year). For the relative rate of changes in SOS%/year, the value was smaller (*p* < 0.001) in the male subjects (0.95%/year) than in the female subjects (1.39%/year).

### 3.2. GAM Analysis of Dietary Vitamin A, C, and E Intakes and Changes in Bone Parameters 

**Figure 1:**
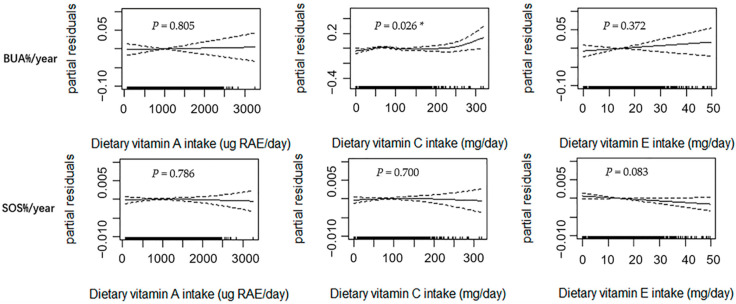
Relationships between dietary vitamin A, C, and E intakes and annual relative rates of changes in BUA and SOS estimated using the generalized additive model (GAM) among all subjects (*n* = 1418). Notes: Age, sex, weight, height, dietary energy intake, dietary vitamin D intake, dietary calcium intake, physical activity, pubertal stage, and baseline bone phenotype were adjusted in the model. The y-axis is the partial residuals of changes in bone parameters after removing the effects of the covariates. The x-axis is the estimated dietary vitamin A, C, and E intakes, and the rug plot along the bottom represents each observation. The solid line indicates the fitted curve between variables. The dashed lines represent the 95% confidence intervals. RAE, retinol activity equivalents; BUA%/year, annual relative rate of change in broadband ultrasound attenuation; SOS%/year, annual relative rate of change in speed of sound; * p < 0.050.

Figure 1 shows the results of the GAM analysis among all subjects (*n* = 1418), with adjustments made for the confounding factors of age, sex, weight, height, dietary energy intake, dietary vitamin D intake, dietary calcium intake, physical activity, pubertal stage, and baseline value of bone phenotype. BUA%/year exhibited a curvilinear relationship with dietary vitamin C intake (*p* = 0.026). When the dietary vitamin C intake value was greater than the inflection point, a positive relationship was noted between the two components. Although linear upward trends were observed between dietary vitamin A and E intakes and BUA%/year, the trends were not statistically significant (*p* > 0.050). A marginally significant linear association was observed between dietary vitamin E intake and SOS%/year (*p* = 0.083). No significant relationship was observed between dietary vitamin A and C intakes and SOS%/year (*p* > 0.050). 

**Figure 2:**
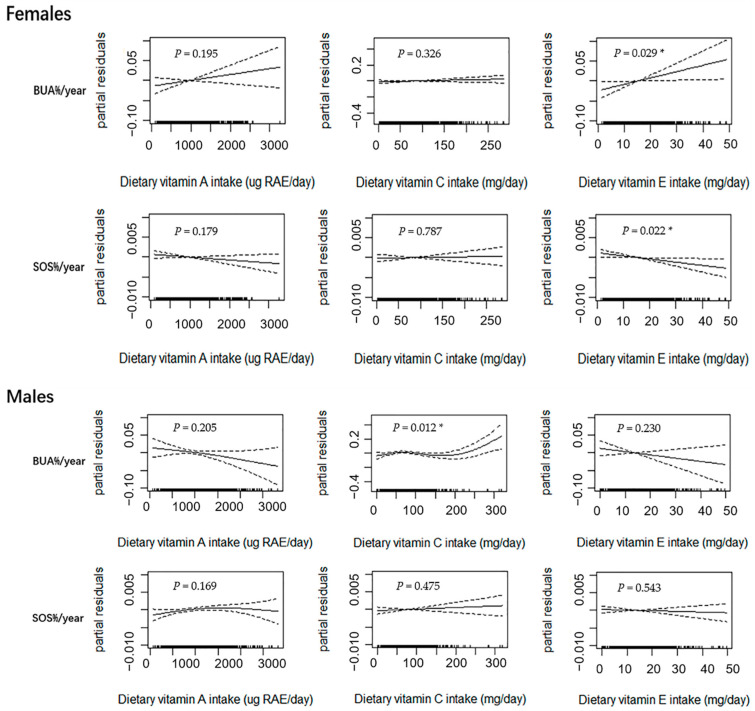
Relationships between dietary vitamin A, C, and E intakes and annual relative rates of change in BUA and SOS estimated using the generalized additive model (GAM) in female (*n* = 721) and male (*n* = 697) adolescents. Notes: Age, weight, height, dietary energy intake, dietary vitamin D intake, dietary calcium intake, physical activity, pubertal stage, and baseline bone phenotype were adjusted in the model. The y-axis is the partial residuals of changes in bone parameters after removing the effects of covariates. The x-axis is the estimated dietary vitamin A, C and E intakes, and the rug plot along the bottom represents each observation. The solid line indicates the fitted curve between variables. The dashed lines represent the 95% confidence intervals. RAE, retinol activity equivalents; BUA%/year, annual relative rate of change in broadband ultrasound attenuation; SOS%/year, annual relative rate of change in speed of sound; * p < 0.050.

Figure 2 shows the results of the GAM subgroup analysis in the female (*n* = 721) and male (*n* = 697) subjects after the adjustment of age, weight, height, dietary energy intake, dietary vitamin D intake, dietary calcium intake, physical activity, pubertal stage, and baseline bone phenotype. In the female subjects, the dietary intake of vitamin E was linearly correlated with BUA%/year (*p* = 0.029) and SOS%/year (*p* = 0.022). No other significant associations were observed, although there was a linear upward trend between dietary vitamin A intake and BUA%/year in the female group (*p* > 0.050). In the male subjects, a curvilinear relationship for dietary vitamin C intake and BUA%/year was observed (*p* = 0.012) with an inflection point. This was similar to the results among all subjects. Although linear or near-linear downward trends between dietary vitamin A and E intakes and BUA%/year were observed, the trends were not statistically significant (*p* > 0.050). No significant relationships were observed between dietary vitamin A, C, and E intakes and SOS%/year (*p* > 0.050).

### 3.3. Linear Regression Analysis for the Association between Dietary Vitamin E Intake and Changes in Bone Parameters 

Multiple linear regression analysis was performed to estimate and infer the associations between dietary vitamin E intake and BUA%/year and SOS%/year. Dietary vitamin E intake was positively associated with BUA%/year (sβ = 0.082, *p* = 0.033) in the female subjects (Table 2). No other significant relationships were observed overall o among the male subjects in terms of dietary vitamin E intake and BUA%/year and SOS%/year (*p* > 0.050). 

### 3.4. Threshold Analysis for the Dietary Vitamin C Intake and Changes in BUA in Male Subjects 

According to the GAM analysis, the association of dietary vitamin C intake with BUA%/year was curvilinear in the male subjects. Subsequently, a two-piecewise multiple linear regression analysis was carried out to detect the potential threshold effects of dietary vitamin C intake on BUA%/year. The results showed that the break-point of dietary vitamin C intake was 159.01 mg/day in the male subjects; BUA%/year was not significantly related to the dietary intake of vitamin C below this threshold (sβ = −0.016, *p* = 0.666) (Table 3). When the dietary vitamin C intake was greater than 159.01 mg/day, a positive and significant association with BUA%/year (sβ = 0.395, *p* = 0.036) was noted after the adjustment of age, weight, height, dietary energy intake, dietary vitamin D intake, dietary calcium intake, physical activity, pubertal stage, and baseline BUA.

## 4. Discussion

In this study, we detected associations of dietary vitamin A, C, and E intakes with the changes in bone parameters in 1418 Chinese adolescents over a 2.5-year follow-up period. The results indicated a curvilinear relationship between the dietary intake of vitamin C and BUA%/year for all the subjects. Further subgroup analyses revealed that the curvilinear relationship was observed in the male adolescents and indicated a potential threshold effect. In addition, dietary vitamin E intake was linearly positively related to BUA%/year in the female adolescents. No association was found between dietary vitamin A intake and the annual relative rates of changes in bone parameters.

A curvilinear relationship was observed between dietary vitamin C intake and bone mass accumulation in the male adolescents. Data from this study showed that bone mass gained with the increase of dietary vitamin C intake only in the boys with a dietary vitamin C intake value approximately > 159 mg/day. The results of a study conducted on 663 Japanese adolescents aged 8–14 years corroborated this finding [16]; the authors reported that the dietary intake of vitamin C was positively associated with bone area ratio only in boys with a dietary vitamin C intake greater than the recommended dietary allowance (>65 mg/day) [16]. Previous studies in adult men have also indicated a nonlinear relationship of dietary vitamin C intake with bone phenotype [26,27]. In a cohort of 213 men aged 28–62 years, a significantly lower bone loss rate at the trochanter and lumbar spine was observed only in those with the highest tertile of dietary vitamin C intake (approximately > 200 mg/day) [26]. Simon et al. studied 6137 men aged 20–90 years and reported that a dietary vitamin C intake of approximately 100–250 mg/day reduces the probability of fractures but that this probability increases in those with an intake of <100 mg/day or >250 mg/day [27]. These studies might indicate that an appropriate intake of vitamin C beyond the recommended ranges may help gain and maintain bone mass. Studies showed that eating more fruits and vegetables was associated with increased bone mass, probably because fruits and vegetables are rich in antioxidant nutrients, such as vitamin C [14]. The antioxidant effects of vitamin C were discussed in the background of this article. In addition to vitamin C’s being an antioxidant with benefits to human bone health [10,11], it is also essential for the synthesis and maturation of type I collagen [28], which is the major component of bone matrix [29]. Vitamin C also participates in osteoclastogenesis and osteoblastogenesis. An animal study reported that vitamin C-deficient mice exhibited the elevated expression of receptor activator of nuclear factor-kappa B ligand, which acts as a critical osteoclast and bone resorption inducer [30]. Moreover, the expression of peroxisome proliferator-activated receptor isoform γ in osteoblasts was significantly increased in the vitamin C-deficient mice [30]. Other studies revealed that peroxisome proliferator-activated receptor isoform γ may suppress osteoblast differentiation and bone formation [31].

Our study suggested that dietary vitamin E can contribute to bone mass gain in female adolescents. The findings are inconsistent with those reported in the only study conducted in adolescents [16]. The cross-sectional study involving 333 Japanese girls aged 8–14 years observed that the dietary intake of vitamin E was not related to bone area ratio [16]. However, our study findings were similar to the results of some other previous studies conducted in adults [32,33,34]. Odai et al. studied 157 Japanese women aged 38–76 years and found that the dietary intake of α tocopherol had a positive correlation with BMD [32]. Chan et al. observed that the dietary intake of vitamin E was positively related to spinal BMD in 441 Chinese females aged 20–35 years [33]. Shi et al. studied 2178 Chinese women and 1025 Chinese men aged 40–75 years and reported that the dietary intake of vitamin E was correlated with a larger BMD value at various bone sites in women but not in men [34]. The mechanism underlying the action of vitamin E on the bone may be as follows: as an antioxidant, vitamin E protects the bones by generating ROS and improving the body’s antioxidant status [10,11]. Moreover, studies reported that vitamin E inhibited osteoclast formation and maturation by inhibiting the expression of receptor activator of nuclear factor-kappa B ligand and N-terminal telopeptide and by promoting the expression of osteoprotegerin [35]. Cytokines are also major factors in regulating inflammatory responses in metabolic bone diseases. Vitamin E reportedly affects bone health by inhibiting the excessive levels of inflammatory factors such as tumour necrosis factor-alpha, interleukins, and interferons [36]. Vitamin E also helps maintain bone health by promoting the expression of bone-related growth factors such as insulin-like growth factor 1, bone morphogenetic protein, and vascular endothelial growth factor [9,37] and by regulating the levels of bone metabolic hormones such as insulin and leptin [38]. 

Dietary vitamin A intake was not related to the bone parameters assessed in this study; several studies have also investigated this association among adolescents and have reported similar results. A cross-sectional study of 321 Norwegian adolescents aged 11–17 years reported that dietary vitamin A intake had no significant association with forearm BMD [12]. Kohri et al. studied 663 Japanese individuals aged 8–14 years and observed that the dietary intake of vitamin A was positively related to bone area ratio among premenarcheal females but not among males or postmenarcheal females [16]. In our study, approximately 89% of the female subjects were postmenarcheal. In adult studies, the correlation between dietary vitamin A intake and bone parameters was complicated [39,40]. A study involving individuals aged 55–92 years observed a U-shaped relationship between dietary retinol intake and bone mass [41]. Another study reported that a dietary retinol intake of >1500 µg/day resulted in a low BMD and increased the risk of osteoporotic fractures [42]. Our study then also analysed these data in adolescents whose dietary vitamin A intake values exceeded the recommended ranges (females > 630 μg RAE/day; males > 670 μg RAE/day) but observed no correlation between dietary vitamin A intake and the bone parameters. Some studies have also reported that the negative associations observed [41,42] were attributable to not only the excessive vitamin A intake but also the amount of retinol-fortified foods consumed (low fat/skim milk). Taking vitamin A supplements as an aqueous dispersion or emulsion rather than an oil suspension yields a higher vitamin A peak in plasma [43]; this may be because ingesting retinol in water form rapidly increases the retinol concentrations in the intestinal mucosa. When the absorption is rapid, the retinol-binding proteins may be temporarily saturated, resulting in the oxidation of retinol to all-trans-retinoic acid [39]. Excess retinoic acid in the body accelerates bone resorption [44]. In this study, vitamin A was mainly obtained from ordinary food, i.e., liver foods. This might be the reason why no relationship was found between the dietary vitamin A and the bone parameters even in the subjects in whom the intake value exceeded the recommended ranges. 

There could be sex differences in the relationships of dietary vitamin E and C intakes with bone mass gain in adolescents. Vitamin E, a fat-soluble vitamin, mainly comes from vegetable oils, grains, nuts, and seeds [45], and it is mainly deposited in adipose tissue [46]. Females generally have more fat mass than males at the same body mass index (BMI) [47]. In this adolescent population, the BMI in girls was higher than that in boys. Thus, females could store more vitamin E than males in body. In addition, compared with the girls, the dietary vitamin E intake in boys was lower in this study. These could limit the finding of a beneficial association between dietary vitamin E intake and changes in bone parameters in boys. For dietary vitamin C intake, no significant association was found in girls. This was consistent with the results from some other studies in children and adolescents [14,15,16]. A cross-sectional study involving 257 subjects aged 16–18 years in the United States found that dietary vitamin C intake was positively associated with bone mass in boys but not in girls [14]. Another study conducted in 663 Japanese adolescents aged 8–14 years also observed that dietary vitamin C intake was positively related to bone area ratio in the male group and not in the premenarcheal and postmenarcheal female groups [16]. In this study, most of the studied female subjects (~89%) were postmenarcheal. The levels of estrogens (e.g., estradiol) increase during puberty and may affect bone mass gain [48]. It is possible that the effects of estrogen could mask the weak influence of dietary vitamin C intake on bone mass gain in female adolescents. Nonetheless, the serum estradiol level was not significantly correlated with bone mass gain in the girls of this population [49]. Moreover, studies also revealed sex differences in the associations between some other nutritional factors and bone mass, i.e., calcium, phosphorus, magnesium, and vitamin D [50,51]. The sex differences may be attributed to the different nutrients required for bone mass accumulation between female and male adolescents. Related studies in adolescents were limited, and more research is needed to explore this difference.

To the best of our knowledge, this is the first longitudinal study to report the effects of dietary vitamin A, C, and E intakes on the changes of bone parameters in adolescents. However, there were also some limitations in our study. First, the bone parameters were measured by QUS, not the gold standard DXA, although QUS-derived bone parameters have been shown to be well associated with those measured by DXA [18]. Second, as only the dietary intake data at baseline were used in this study, the results may be attenuated by possible changes in diet over time and random error from a single measurement. Nonetheless, the subjects in this study were all school students who ate meals in the school canteen for most of the year. To some extent, the baseline dietary intake data might reflect the dietary intake of the subjects in the 2.5 years of follow-up. Third, the calcium and vitamin D from dietary intakes were estimated and added to the model as covariates but not considering those from supplements use and the vitamin D derived from sunlight exposure. Calcium and vitamin D play important roles in bone metabolism [52,53]. Calcium is a major component of human bone tissue [54]. Vitamin D is essential for the calcium homeostasis and mineralisation within bones [54]. This study may have underestimated the calcium and vitamin D levels of the subjects. Nonetheless, only around 3.95% of the subjects (29 females and 27 males) were taking supplements, particularly multivitamins. For the sunlight exposure time, the differences could be moderate or mild among the students, as their exposure time to the sunlight is mainly from the outdoor physical education classes and sports activity time. In addition, the outdoor exercise time could reflect the sunlight exposure time to some extent. Its correlations with bone parameters were weak (*r* < 0.05) and not significant in these students. Fourth, the 2.5 year follow-up duration might not be sufficient for assessing the changes in bone architecture. In this study, the relative rate of the change in BUA varied by approximately 21% per year, whereas the relative rate of change for SOS was only 1% per year. Finally, all the individuals were recruited from only one secondary school. This may restrict the generalisability of our study findings. 

## 5. Conclusions

In conclusion, dietary vitamin C intake was observed to have a threshold effect on bone mass gain in the male adolescents, and dietary vitamin E intake was observed to be a positive predictor for bone mass gain in the female adolescents. Our findings may promote the general understanding of the influence of antioxidant vitamins on bone mass accumulation in Chinese adolescents and may provide useful information for the establishment of a model for predicting PBM.

## Figures and Tables

**Table 1:** Basic characteristics of the studied subjects.

Values	Total (*n* = 1418)	Girl (*n* = 721)	Boy (*n* = 697)	*p*
Age (years)	14.97 ± 1.46	14.99 ± 1.47	14.95 ± 1.45	0.974
Height (cm)	161.34 ± 9.33	157.54 ± 5.85	165.27 ± 10.56	<0.001 *
Weight (kg)	49.99 ± 10.59	47.72 ± 8.13	52.33 ± 12.20	<0.001 *
Total physical activity (MET·h/day)	19.59 ± 9.60	19.44 ± 10.45	19.75 ± 8.64	0.007 *
Dietary energy intake (kcal/day)	2185 ± 845	1909 ± 827	2471 ± 764	<0.001 *
Dietary calcium intake (mg/day)	396.90 ± 171.18	356.41 ± 149.60	438.78 ± 181.81	<0.001 *
Dietary vitamin D intake (µg/day)	1.85 ± 1.29	1.81 ± 1.14	1.90 ± 1.43	0.467
Dietary vitamin A intake (µg RAE/day)	994.10 ± 528.45	973.10 ± 535.78	1015.82 ± 520.26	0.052
Dietary vitamin C intake (mg/day)	82.57 ± 47.37	84.81 ± 48.93	80.25 ± 45.62	0.118
Dietary vitamin E intake (mg/day)	14.59 ± 8.64	15.33 ± 8.48	13.82 ± 8.74	<0.001 *
Annual relative rate of BUA change (%/year)	20.50 ± 16.93	18.92 ± 16.19	22.13 ± 17.53	0.002 *
Annual relative rate of SOS change (%/year)	1.17 ± 0.81	1.39 ± 0.81	0.95 ± 0.75	<0.001 *

Notes: Data are presented as mean ± standard deviation. MET, metabolic equivalent; RAE, retinol activity equivalents; BUA, broadband ultrasound attenuation; SOS, speed of sound; * *p* < 0.050.

**Table 2:** Results of associations between dietary vitamin E intake and annual relative rates of change in BUA and SOS in the linear regression analyses.

	BUA%/year		SOS%/year	
	sβ	*p*	sβ	*p*
Total (*n* = 1418)	0.027	0.331	−0.036	0.184
Females (*n* = 721)	0.082	0.033 *	−0.065	0.092
Males (*n* = 697)	−0.042	0.283	−0.014	0.736

Notes: Age, weight, height, dietary energy intake, dietary vitamin D intake, dietary calcium intake, physical activity, pubertal stage, and baseline bone phenotype and/or sex were adjusted in the analysis. BUA%/year, annual relative rate of change in broadband ultrasound attenuation; SOS%/year, annual relative rate of change in speed of sound; sβ, standardized regression coefficient; * *p* < 0.050.

**Table 3:** Threshold analysis of dietary vitamin C intake and annual relative rate of change in BUA in males (*n* = 697).

Dietary Vitamin C Intake	BUA%/year	
	sβ	*p*
<159.01 (mg/day)	−0.016	0.666
≥159.01 (mg/day)	0.395	0.036 *

Notes: Age, weight, height, dietary energy intake, dietary vitamin D intake, dietary calcium intake, physical activity, pubertal stage, and baseline BUA were adjusted in the analysis. BUA%/year, annual relative rate of change in broadband ultrasound attenuation; sβ, standardized regression coefficient; * *p* < 0.050.

## Data Availability

Requests for data may be directed to the corresponding author and are subject to institutional data use agreements.
